# Transcriptional regulators of arterial and venous identity in the developing mammalian embryo^☆^

**DOI:** 10.1016/j.cophys.2023.100691

**Published:** 2023-10

**Authors:** Ian R McCracken, Andrew H Baker, Nicola Smart, Sarah De Val

**Affiliations:** 1 Institute of Developmental and Regenerative Medicine, Department of Physiology, Anatomy and Genetics, University of Oxford, Oxford OX3 7TY, United Kingdom; 2 Centre for Cardiovascular Science, University of Edinburgh, Edinburgh EH16 4TJ, United Kingdom

## Abstract

The complex and hierarchical vascular network of arteries, veins, and capillaries features considerable endothelial heterogeneity, yet the regulatory pathways directing arteriovenous specification, differentiation, and identity are still not fully understood. Recent advances in analysis of endothelial-specific gene-regulatory elements, single-cell RNA sequencing, and cell lineage tracing have both emphasized the importance of transcriptional regulation in this process and shed considerable light on the mechanism and regulation of specification within the endothelium. In this review, we discuss recent advances in our understanding of how endothelial cells acquire arterial and venous identity and the role different transcription factors play in this process.

**Current Opinion in Physiology** 2023, **35**:100691

This review comes from a themed issue on **Endothelium**

Edited by **Jeremy Pearson and Paul C Evans**

For complete overview of the section, please refer to the article collection, “Endothelium”

https://doi.org/10.1016/j.cophys.2023.100691

2468–8673/© 2023 The Author(s). Published by Elsevier Ltd. This is an open access article under the CC BY license (http://creativecommons.org/licenses/by/4.0/).

## Introduction

The blood vasculature is a closed network of interconnected arteries, capillaries, and veins that together function to transport gases, nutrients, cells, proteins, and signaling molecules throughout the body. Endothelial cells (EC) comprise the innermost layer of all blood vessels, providing a barrier between blood and vessel wall while responding to an array of different physical and chemical signals to maintain vascular homeostasis [1]. The formation of new blood vessels occurs using either vasculogenesis (deriving ECs from mesodermal progenitors during initial vascular formation) or angiogenesis (deriving ECs from existing vessels via sprouting or intussusception) [2]. In addition to the formation of new vessels, the growth and maturation of the vascular system also require that they adapt into an arterial–capillary–venous network [3]. This results in a highly heterogeneous endothelium consisting of ECs with significant physical and functional differences [3], responding to both cell-intrinsic signals and information from the local environment.

## Formation of the mature arteriovenous network

### Origins of arterial and venous endothelial cells

**Figure 1:**
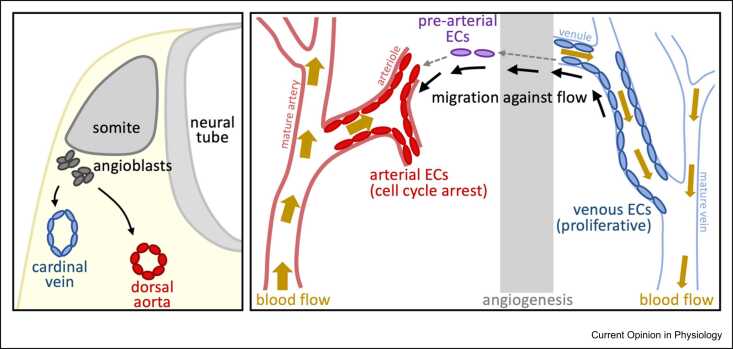
Schematic summary of different methods of arterial formation. **(a)** summarizes arteriovenous differentiation during early embryogenesis when EC were formed by vasculogenesis, whereas **(b)** summarizes the vein-to-arterial transition endothelial transition that can be seen during later embryonic timepoints.

Adapted from Red-Horse and Siekmann (2019) BioEssays [6]; and Siyuan et al (2022) Cell Research [80].

The first arteries and veins form during vasculogenesis, with EC progenitors first aligning to form the paired dorsal aorta and venous structures arising slightly later in distinct locations (Figure 1) 4, 5•, 6. Although many classical arteriovenous markers are not detected during these early stages, EC progenitors with activated Notch signaling are associated with arterial identity [4].

Formation and growth of arteries and veins at later timepoints were originally thought to follow that of other tubular organs, with new arteries and veins budding outward from existing ones. However, overwhelming evidence from fate-mapping and single-cell RNA sequencing (scRNA-seq) analysis now strongly indicates that venous ECs are the primary source of both angiogenic and arterial ECs, with new arteries forming via inward growth and coalescence of newly differentiated arterial ECs from a venous/capillary origin [6]. Mammalian examples of this vein-to-arterial EC differentiation include embryonic coronary vessels (differentiation of arterial ECs from sinus venosus) 7, 8•, mid-gestation mice and similarly staged human embryos (arterialization of capillary ECs with venous characteristics) [5], and the postnatal mouse retina (venous EC proliferation, migration, and differentiation into arterial ECs) 9, 10, 11•.

### Influence of blood flow

The initial formation of arterial and venous structures occurs before, and independent of, the initiation of blood flow (e.g. 4, 12). However, full arterial differentiation is not seen in embryos lacking circulation, indicating that blood flow-induced shear stress is necessary for the establishment and/or maintenance of arterial identity 4, 12. Although the mechanisms by which shear stress influences EC fate are still unclear, endothelial Notch1, a key regulator of arterial identity (see below) can act as a mechanosensor of shear stress 13, 14, 15. Blood flow also plays an important role in directing migration during vein-to-arterial EC differentiation, with venous-origin, arterial-fated ECs becoming polarized against the direction of flow and in the direction of their migration 6, 11•, 16.

### Cell cycle state

Recent studies have indicated an important role for cell cycle in arteriovenous identity, with decreased EC proliferation and cell cycling coinciding with the onset of vein-to-arterial EC differentiation 7, 13, 17••, 18•. Arterial-associated levels of fluid shear stress can promote expression of both arterial genes (e.g. *Cx37(Gja4)*) and cell cycle inhibitors [13], while vein-to-arterial differentiation in embryonic coronary vessels was accompanied by a decrease in cell cycle gene expression [7]. Additionally, increased VEGFA and Notch signaling in capillaries can promote arterial EC identity by suppressing MYC-dependent cell cycle progression in both coronary vessels and postnatal retinal vessels [17]. The link between cell cycle state and arteriovenous identity was further confirmed in postnatal retina, where ECs in early G1 were enriched in veins, while ECs in late G1 were enriched in arteries [18]. These cell-state signatures correlate with an enrichment for vein-associated BMP signaling and arterial-associated TGF-β signaling (see below), suggesting that cell cycle state may provide a window of opportunity for molecular inducers of arteriovenous identity to act. However, EC in all states can be found across all EC subtypes [19].

### VEGFA–VEGFR2 signaling

In addition to an essential role during vasculogenesis and angiogenesis, VEGFA signaling is also associated with arteriovenous specification. In general, low VEGFA levels are associated with venous identity, while high concentrations of VEGFA are associated with arterial differentiation and are required for early arterial fate via activation of Notch–RBPJ signaling 20, 21, 22. The best-characterized mechanism by which VEGFA influences arterial identity is VEGFA–VEGFR2-stimulated activation of the MAPK/ERK pathway [23]. In contrast, low VEGFA can induce AKT activation via phosphatidylinositol-3-kinase (PI3K), with PI3K/AKT signaling associated with venous identity 23, 24. VEGFA–VEGFR2 signaling is also implicated in later vein-to-arterial differentiation via the activation of CXCR4–CXCL12 signaling (specific to arterial ECs) and/or the induction of angiogenesis 9, 10.

## Transcription factors associated with arteriovenous gene expression

**Figure 2:**
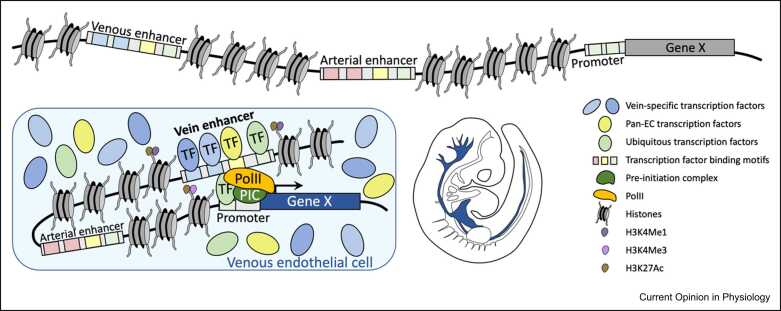
Schematic overview of enhancer function in mammalian cells. Spatial and temporal control of mammalian gene expression is often primarily regulated by enhancers, which are concentrated groupings of TF-binding motifs. A single gene is often regulated by multiple different enhancers, each responsible for different aspects of gene expression. In cells in which the gene is expressed, the correct array of nuclear TFs binds their cognate motifs within the relevant enhancer, which contacts the gene promoter and initiates transcription.

Adapted from Oudelaar and Higgs (2021) Nature Reviews Genetics; [81] and Shlyueva, Stampfel, and Stark (2014) Nature Reviews Genetics [82].

**Figure 3:**
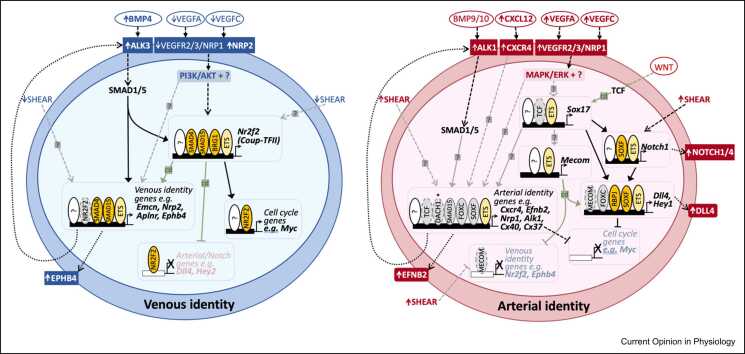
Schematic overview of the regulatory pathways governing gene expression and their interaction with transcriptional regulators in venous and arterial EC. Vertical ovals denote the TFs regulating the named gene(s). Yellow coloring indicates that the designated TF is confirmed to directly bind a characterized and verified enhancer/promoter, solid gray coloring indicates strong evidence for direct transcriptional link but no verified enhancer/promoter binding, and dashed lines of oval indicate a TF that has been linked due to gene expression changes after altered expression. Solid arrows indicate direct transcription link, dashed arrows indicate indirect link. Black-colored lines represent known links, gray lines (with a ?) represent hypothesized links, and green lines (with cd) indicate where there is conflicting data on the link. Horizontal ovals indicate growth factors, horizontal squares indicate receptors, and horizontal rounded squares indicate ligands. The ETS oval generally indicates any EC-expressed member of the ETS TF family, although in most cases, both ETS1 and ERG have been documented directly binding at the locations indicated. Asterisk * over DACH1 indicates that protein is not found in mature arterial ECs.

Arterial versus venous EC identity is associated with significant differences in mRNA expression patterns, indicating an important role for transcriptional regulation. Transcription factors (TFs) play a key role in this process, influencing gene transcription by directly binding specific DNA motifs within cis-regulatory elements (enhancers and promoters, see Figure 2). TFs can bind enhancers and promoters in an array of different combinations alongside other co-activator proteins and downstream of signal pathway-mediated protein modifications, allowing multiple upstream signals to collectively influence patterns of gene expression. Figure 3 provides a schematic overview of what we currently understand about this process.

## ETS transcription factors

Among the numerous TFs involved in endothelial development and identity, none have a more central role than those of the ETS family. ETS proteins are characterized by an ETS domain mediating binding to a core GGA^A^/_T_ motif [25]. All characterized endothelial enhancers and promoters contain multiple ETS-binding domains, and ETS factor binding has been an essential requirement for activity where investigated [2]. Additionally, combinatorial binding of ETS alongside forkhead box (FOX) factors at a composite FOX:ETS motif (AA^C^/_T_AGGA^A^/_T_) is a common feature of many EC-active enhancers and promoters (including pan-EC and arterial EC elements) [26].

Of the 18–20 different ETS factors expressed in ECs at some stage of development, ERG, FLI1, ETS1, ETS2, and ELK3 are the highest expressed and most EC-specific in the mature endothelium, while early embryo-restricted ETV2 plays an essential role in vasculogenesis (reviewed in detail in [2]). Of these, ERG has been most closely investigated for a role in arteriovenous identity, although it is expressed in all ECs [27]. EC deletion of ERG results in vascular defects and embryonic lethality, while induced EC deletion in postnatal retina reduced vascular sprouting, coverage, and density [27]. These phenotypes were linked to roles upstream of both Wnt/β-catenin and Notch signaling, two pathways with arteriovenous links (see below) 27, 28. ERG is also required for activity of an arterial-expressed enhancer for the Notch ligand *Dll4* (Dll4in3) and ETS motifs are found in all other known arterial enhancers (see [2]), leading to the hypothesis that ERG directly regulates arterial specification downstream of VEGFA/MAPK/ERK signaling 29, 30. However, pan-vascular, angiogenic-specific, and vein-specific enhancers also contain multiple ETS-binding motifs and can experience increased ERG binding downstream of VEGFA induction, and genome-wide analysis of ERG binding highlights a diverse array of directly targeted EC genes 2, 31•, 32, 33. Further, compound deletion of ERG alongside closely related FLI1 resulted in a complete loss of EC fate and vascular integrity, suggesting an essential requirement for general endothelial identity [34]. Of note, the ERG-bound arterial Dll4in3 enhancer also binds other TFs implicated in arterial development (including RBPJ, SOXF, and FOX) [30]. Consequently, while ERG appears to constitute an essential part of an arterial-specifying TF collective, it may primarily act to promote general endothelial identity.

## RBPJ transcription factor

The TF RBPJ (CSL, suppressor of hairless, LAG1) acts as the nuclear effector of the Notch signaling pathway via directly binding DNA at a consensus TGGGAA motif [25]. In ECs, binding of ligand (e.g. DLL4) to NOTCH1/4 receptors results in nuclear entry of the Notch intracellular domain, which binds to RBPJ alongside the coactivator MAML, allowing it to activate gene transcription [35]. Disruption of Notch signaling in both mouse and zebrafish results in severe arteriovenous defects and a reduction/loss of arterial marker expression (recently reviewed by [36]). This resulted in a model in which NOTCH–RPBJ regulates arterial identity through the direct transcriptional activation of key arterial genes (e.g. [37]), a model strengthened by identification of RBPJ-binding motifs within arterial-specific enhancers for *Dll4* and *Hey1* 2, 30, 38. However, these observations come with a number of caveats: the RBPJ motif within the arterial and angiogenic-expressed Dll4in3 enhancer (so named as it is located within the third intron of *Dll4*) is not required for arterial activation, except when neighboring SOXF motifs are also ablated; both *Dll4* and *Hey1* are themselves components of NOTCH signaling; enhancers for other arterial-specific genes (e.g. *Ece1*, *Notch1*) do not feature obvious RBPJ motifs 30, 39, 40. Crucially, it has now been shown that arterial gene transcription and identity still occur in the absence of RBPJ providing that MYC is also deleted [17]. These results strongly indicate that the principal role of Notch–RPBJ in arterial differentiation is to reduce metabolism and cell cycle rather than directly activating transcription of arterial identity genes 17••, 19•, 41•.

## SOXF transcription factors

Members of the SOX family (divided into subgroups A–S) are characterized by a conserved HMG box binding a consensus ^A^/_T_CAA^A^/_T_ DNA motif [25]. The SOXF subgroup (SOX7, SOX17, and SOX18) are the predominant SOX factors in ECs, where they play important roles in vasculogenesis, angiogenesis, and lymphatic specification (see 2, 42). Additionally, arterial enhancer analysis and gene ablation studies firmly link SOXF factors with arterial identity. SOX-binding motifs are a common feature of the limited number of well-characterized arterial enhancers while not found within the few characterized vein-specific enhancers (see [2]). However, the requirement for SOXF varies: SOX-binding motifs are essential for activity of *Notch1* and *Ece1* but not required for *Flk1* and *Dll4* enhancers 30, 40, 43, 44. Mutant animal models also support a role for SOX17 in arterial identity, although varying levels of genetic compensation between SOXF factors on different mouse backgrounds complicate analysis 45, 46. Induced deletion of *Sox17* in coronary ECs before arterial specification resulted in abnormal artery formation [47], while embryonic EC deletion of *Sox17* led to arteriovenous shunts, ectopic expression of venous markers in arteries and vice versa, and failure of arterial maturation [48]. However, an initial arteriovenous network is still established in all *Sox17* and compound *Sox17;Sox18* null mice 48, 49. Additionally, although the effect of induced EC deletion of *Sox17* in the postnatal retina varies on different backgrounds, arteriovenous specification still occurred to some degree in all models 46, 48, 50. An essential role for SOX17 in arterial specification/identity may potentially be missed due to compensation from SOX7 and SOX18: *Sox7* is upregulated after *Sox17* deletion, while *Sox7* and *Sox17* compensate for loss of *Sox18* on a mixed mouse background 45, 46. Unfortunately, induced compound EC deletion of *Sox7;Sox17;Sox18* in the postnatal retina results in such severe vascular defects that problems in arteriovenous differentiation cannot be reliably separated from angiogenic defects and loss of flow [46].

While *Sox17* expression is enriched in mature arterial ECs in mammals, *Sox7* and *Sox18* are expressed relatively uniformly across the endothelium [48]. It is therefore unclear how SOX17 could achieve specific activation of arterial gene transcription, as all SOXF factors appear to recognize the same consensus motif. Additionally, SOX17 is not always restricted to pre-arterial ECs at the timepoints that arterial identity is acquired (e.g. it is found in nearly all coronary ECs at the stage where individual pre-arterial ECs emerge 7, 47, 51). It is therefore probable that additional TFs are required alongside SOXF factors to specifically activate arterial gene transcription. For example, arterial activity of the Dll4in3 enhancer was lost only after both SOXF and RBPJ motifs were mutated [30].

## DACH1

DACH1 is a widely expressed helix–turn–helix TF with a currently poorly defined consensus DNA-binding motif. During embryonic coronary development, DACH1 is enriched in ECs in capillaries and vessels undergoing initial arterial remodeling, but was not found in larger arteries, an expression pattern shared in the postnatal retina and linked to shear stress [52] In embryonic coronary vessels (the vascular bed in which it has been primarily studied), both global and EC *Dach1* deletion resulted in smaller coronary arteries with reduced lumen size, a phenotype associated with reduced expression of *Cxcl12*, and decreased EC polarization against flow. Conversely, overexpression of *Dach1* from E13.5 resulted in increased numbers of coronary arterial ECs (as marked by CX40 (GJA5)), while a similar experiment in postnatal retina resulted in an increased arterial branch length [41]. ScRNA-seq analysis in these models suggests that DACH1 potentiates normal arterial differentiation, rather than acting as a master regulator of arterial EC identity, in agreement with the fact that mice without EC *Dach1* can survive to adulthood 41•, 52.

## MECOM

MECOM has recently been identified as one of the most differentially expressed arterial TFs in mammals 5•, 7, 19•. MECOM is a member of the PRDM family of lysine methyltransferases and has been linked to methylation of both H3K9 (associated with transcriptional repression) and H3K4 (transcriptional activation), in addition to directly binding DNA via zinc finger domains [53]. Consequently, it can act as both a TF and epigenetic regulator. Confusingly, the gene currently known as *Mecom* covers several splice variants previously annotated as *Mds1* and *Evi1* [53] (Mecom stands for Mds1 and Evi1 complex locus). As such, it has been primarily studied as an oncogene contributing to leukemogenesis, although it is also implicated in a variety of developmental processes [53]. *Mecom* mutant mice lacking all three *Evi1* isoforms die between E13 and E16 [54], while an alternative *Mecom* mutant model ablating only two *Evi1* isoforms reported earlier lethality at E10.5 [55]. In both cases, defective vasculature and hemorrhage were observed, but the vasculature was not closely investigated. Analysis of MECOM function in mammalian ECs has therefore primarily been restricted to siRNA-mediated knockdown in a human embryonic stem cell-derived arterial EC model, which resulted in increased transcription of venous and lymphatic genes, while expression of arterial-associated Notch genes was unaffected [19]. Conflictingly, analysis of *mecom* in zebrafish reported a similar selective expression in the dorsal aorta, yet morpholino-mediated knockdown resulted in reduced expression of Notch pathway genes, while the venous marker *flt4* was unaffected [56]. It is therefore clear that further analysis of MECOM is required to determine its transcriptional role(s) in determining arterial EC identity.

## Forkhead box transcription factors

FOX proteins form an evolutionary-conserved group of transcriptional regulators involved in a diverse range of functions, directly binding DNA at a core ^C^/_T_AAA^C^/_T_A motif [25]. FOX proteins are grouped into subclasses A–S, with subclasses FOXC and FOXO expressed in ECs. FOXO proteins (FOXO1, 3, and 4) are a core target of P13K/AKT signaling, a pathway associated with venous specification (see above and [57]). In the absence of PI3K/AKT signaling, FOXOs localize in the nucleus, whereas P13K activation results in nuclear exclusion. Mutational models of *FOXO* factors have demonstrated their important roles in vascular development. EC deletion of *Foxo1* results in lethality by E10.5 associated with profound vascular defects and growth retardation, while induced EC deletion in the postnatal retina caused EC hyperproliferation 58, 59. Although EC deletion of *Foxo1* reduced expression of the Notch ligand *Dll4* in the yolk sac vasculature (related to a role for FOXO1 in repressing Spry genes), no clear embryonic arteriovenous defects have been reported [60]. In contrast, FOXC proteins were implicated in arterial identity after compound global *Foxc1;Foxc2* deletion resulted in arteriovenous malformations. Analysis of *Foxc1;Foxc2* mutants found greatly reduced arterial EC gene expression, while expression of venous genes was unaffected [61]. Overexpression of *Foxc1/2* in cultured cells also increased arterial gene expression [61]. This was originally linked to binding of FOXC to the promoters of *Hey2* and *Dll4* [62], although subsequent studies have suggested that these binding regions were not essential for arterial expression [29]. Although FOX motifs were found within arterial-specific enhancers for *Dll4* and *Hey1*, they were not required for arterial activity of these elements 30, 38. Adding to the complexity, both FOXC and FOXO factors bind DNA alongside ETS factors at compound FOX:ETS motifs associated with early pan-EC activity (see ETS section) [26]. Therefore, while FOX proteins may be required for arteriovenous identity, their precise transcriptional targets have still not been clearly elucidated.

## NR2F2 transcription factor

NR2F2 (COUP-TFII) is a widely expressed member of the orphan nuclear receptor superfamily and can bind DNA at nuclear receptor half-sites (e.g. ^A^/_G_ AGGTCA [33]). Unlike many other arteriovenous TFs, *Nr2f2* expression in the endothelium is relatively specific to venous and lymphatic ECs, and is sharply decreased in ECs as they undergo vein-to-arterial differentiation [7]. Deletion of *Nr2f2* in ECs resulted in embryonic lethality by E12 [63]. Although the initial arteriovenous network was formed in these mutants, venous ECs ectopically expressed arterial genes [63]. Conversely, overexpression of NR2F2 suppressed Notch components, leading to a model in which NR2F2 regulated venous identity via repression of Notch signaling [63]. However, scRNA-seq analysis found that while overexpression of *Nr2f2* during coronary vessel differentiation prevented ECs from acquiring arterial fate, it did not alter the expression of Notch genes [7]. Instead, *Nr2f2* overexpression resulted in increased cell cycling and proliferation, leading to a new hypothesis linking *Nf2r2* expression to regulation of cell cycling [7]. Supporting this, NR2F2 binding in ECs was overrepresented around genes associated with cell cycle [33]. Further analysis of NR2F2 binding in arterial and venous ECs suggested that NR2F2 rarely directly inhibited arterial genes, instead primarily binding alongside ETS factors to upregulate vein-associated loci [33]. However, no transgenic-verified enhancers have yet been demonstrated to directly bind NR2F2, complicating analysis of its direct influence on the transcription of arterial and venous genes.

As NR2F2 plays a central role in venous specification, there has also been considerable interest in the regulatory pathways upstream of *Nr2f2* expression. Analysis in zebrafish placed the widely expressed SOX7 and SOX18 TFs upstream [64]. Additionally, PI3K/AKT signaling downstream of Tie2 (the receptor for angiopoietin 1) can increase NR2F2 stabilization in venous ECs [65], whereas the chromatin remodeling enzyme BRG1 also promotes *Nr2f2* expression [66]. Alternatively, analysis of an enhancer for *Nr2f2* (named CoupTFII-965) directing vein-specific expression in both mouse and zebrafish models has instead implicated the Receptor (R)-SMAD TFs SMAD1/5 as direct transcriptional activators of *Nr2f2* downstream of BMP signaling in venous ECs [31].

## Receptor-SMAD transcription factors

R-SMAD TFs (SMAD1–3, 5, and 9) are the principal signal transducers of the TGF-β superfamily of ligands, broadly divided into TGFβ-like and BMP-like ligands. After receptor-mediated activation, R-SMADs move to the nucleus, complex with SMAD4, and activate gene transcription by both direct and indirect DNA binding [67]. In general, TGF-β ligands activate SMAD2/3, and BMP ligands activate SMAD1/5/9, although TGF-β in ECs can differentially activate all R-SMADs depending on receptor (reviewed by 67, 68). Components of TGF-β/BMP signaling have been implicated in both venous and arterial fates. The TGF-β receptor ALK1/ACVRL1 is highly expressed in arterial ECs and loss at embryonic or adult stages results in arteriovenous defects, including the loss of arterial markers [69]. Additionally, activation of the arterial-enriched *Cx37/Gja4* is linked to BMP9–ALK1–SMAD1/5 signaling [70]. Conversely, BMP–ALK3(BMPR1a)–SMAD1/5 signaling is essential for venous identity in the early mouse embryo [31]: SMAD1/5 directly binds essential motifs within vein-specific enhancers for *Nr2f2* and *Ephb4* genes, and EC-specific deletion of either *Alk3* or *Smad4* resulted in defective vein formation, reduced *Ephb4* and *Nr2f2* expression, and embryonic lethality by E10.5 [31]. Further, the ALK2/3 ligand BMP4 is enriched at sites of early vein formation in the embryo, and differentially upregulated in venous EC during coronary vessel differentiation 31•, 41•. Compound EC deletion of *Smad1/5* results in similar-stage embryonic lethality to loss of *Smad4* alongside impaired Notch signaling and angiogenesis defects [71], while EC deletion of *Smad4* in the postnatal retina resulted in arteriovenous malformations 72, 73. However, while ChIP-seq analysis of SMAD1/5 binding in ECs confirmed their direct interaction with venous enhancers, it also found binding at arterial gene loci (e.g. *Cx40*, *Hey1*) 31•, 74, in keeping with a role in both arterial and venous differentiation. While additional TFs may therefore be needed for specificity of SMAD1/5, it has also been proposed that the different cell cycle states of arterial and venous ECs (discussed above) may influence the transcriptional response to BMP and TGF-β signaling [18].

## TCF/LEF transcription factors

The TCF/LEF TFs (TCF7, TCF7L1, TCFL2, and LEF1 in mammals) are the primary transcriptional effectors of canonical WNT/β-catenin signaling. They bind DNA at consensus ^A^/_T_^A^/_T_ CAAAG motifs [25] after complexing with nuclear β-catenin downstream of ligand-receptor binding [75]. Analysis of the EC role for WNT/β-catenin/TCF has produced somewhat conflicting results. EC-specific β-catenin loss and gain of function can result in embryonic lethality alongside defective EC differentiation and altered arteriovenous remodeling [76], however, other studies found only CNS-specific defects after β-catenin perturbation (e.g. [77]). Likewise, mapping the activity of WNT/β-catenin/TCF using WNT reporter alleles such as TOP-gal and BAT-gal (multimerized TCF-binding motifs upstream of a reporter gene) also yielded mixed results. For example, BAT-gal was alternatively reported to be active in ECs throughout the embryo [76] or silent in all ECs [29]. Additionally, no currently characterized arterial- or vein-specific enhancers/promoters contain defined TCF- binding motifs [2]. Despite these limitations, there is some evidence supporting a role for WNT–β-catenin–TCF signaling in arteriovenous identity. Constitutive activation of β-catenin signaling resulted in reduced expression of vein-associated *Ephb4*, expanded activity of arterial-associated *Efnb2*, and upregulation of *Dll4* [76]. Further, activation of Wnt/β-catenin signaling alongside Notch induction resulted in increased *Dll4* expression in cultured cells, although this process was thought to involve the complexing of β-catenin with RBPJ rather than TCF factors, and was mapped to a RBPJ motif in the Dll4in3 enhancer that is not essential for arterial activity 29, 30, 78. WNT/β-catenin/TCF signaling has also been implicated upstream of SOXF factors in arteries 48, 79. However, EC-specific loss of β-catenin does not interfere with the formation of the dorsal aorta or the expression of *Dll4* in E9.5 embryos [29], and neither loss or gain of β-catenin function affected arteriovenous patterning in the postnatal retina [76]. Further, Norrin-deficient retina still strongly expressed arterial SOXF factors including *Sox17*. Consequently, the exact role(s) of WNT/β-catenin/TCF in arteriovenous identity are still unclear.

## Conclusion

Our understanding of the role of different TFs in the regulation of arteriovenous identity has been greatly assisted by an increased ability to selectively modify gene expression in EC. Further, scRNA-seq has now enabled us to easily identify differentially expressed TFs and target genes, while ATAC-seq and ChIP-seq analyses permit the easier identification of potential differentially active regulatory elements. Analysis of the increasing array of arterial- and venous-specific enhancer and promoter regions has also provided additional and much-needed information. However, it is important to understand the limitations of our current knowledge. Not all proteins playing important roles in the transcriptional regulation of arterial and venous gene expression will themselves be differentially expressed and consequently identifiable by scRNA-seq studies, as other factors such as nuclear localization, protein modifications, preferences for binding partners, and competition for binding motifs can all strongly influence the relative gene activation achieved by TFs. In particular, the influence of the tissue environment may significantly impact the manner in which this limited number of TFs can influence gene expression. Further, gene depletion studies alone can overlook important arteriovenous functions for TFs with close orthologs or multiple functions in the endothelium, and not all open-chromatin or protein-bound elements represent a functional enhancer or promoter element. Lastly while arterial and venous-specific enhancers can provide a convenient platform from which to identify lineage-defining TFs, this analysis is limited by our understanding of the DNA-binding motifs recognized by different TFs, and by the fact that such elements may provide information more relevant to the gene that they regulate than to arteries or veins more generally. As such, it is increasingly important that we take into consideration all aspects when studying this subject.

## Declaration of Competing Interest

The authors wish to confirm that there are no known conflicts of interest associated with this publication and there has been no significant financial support for this work that could have influenced its outcome.

## Data Availability

No data were used for the research described in the article.
